# Design and validation of a questionnaire to measure the attitudes of health professionals towards immigrants (AHPI)

**DOI:** 10.3389/fphar.2024.1287536

**Published:** 2024-10-10

**Authors:** Iwona Niewiadomska, Krzysztof Jurek, Beata Dobrowolska, Alina Deluga, Izabela Mamcarz

**Affiliations:** ^1^ Department of Social Psychoprevention, Institute of Psychology, The John Paul II Catholic University of Lublin, Lublin, Poland; ^2^ Department of Sociology of Culture, Religion and Social Participation, The John Paul II Catholic University of Lublin, Lublin, Poland; ^3^ Department of Holistic Care and Nursing Management, Faculty of Health Sciences, Medical University of Lublin, Lublin, Poland; ^4^ Department of Family and Geriatric Nursing, Faculty of Health Sciences, Medical University of Lublin, Lublin, Poland; ^5^ Simulation Laboratory for Patient Safety, Chair of Medical Education, Medical University of Lublin, Lublin, Poland

**Keywords:** healthcare professionals, immigrant patients, attitude, psychometric properties, validation

## Abstract

**Objective:**

The aim of the article is to describe the validation process of a research tool used to measure the intensity and direction of medical personnel’s attitudes towards immigrant patients.

**Design:**

An instrument validation design with a cross-sectional survey was conducted. The validation process was carried out in two phases. In phase 1, the content validity of the tool was analyzed. The competent judges method was used. The reliability of the judges was verified in terms of the consistency of evaluations (the I-CVI index was calculated). In phase 2, the questionnaire was tested among 340 healthcare professionals who have professional contact with immigrants, in terms of its factorial validity (exploratory and confirmatory factor analysis), internal consistency (reliability measured by Cronbach’s alpha coefficient) and absolute stability (measured by the test-retest method).

**Results:**

The research results indicate satisfactory content validity of the tool (I-CVI>0.8). The reliability of the tool measured by Cronbach’s alpha coefficient was high (0.86). The reliability study based on the test - retest method (after 3 weeks) showed high consistency of measurements (0.75). Exploratory factor analysis allowed extracting 1 factor, which explains 55.7% of the variance. The validity of the one-factor solution was confirmed by confirmatory factor analysis. Satisfactory goodness of fit indices were obtained (RMSEA = 0.038; PCLOSE = 0.624; AGFI = 0.966; GFI = 0.990; CFI = 0.996).

**Conclusion:**

The presented tool has satisfactory psychometric properties. The AHPI is a tool that can be used for quick assessment of the intensity and direction of attitudes of medical students and healthcare professionals towards immigrant patients, which can help shape educational and training processes.

## 1 Introduction

The phenomenon of migration affects all countries and people in an era of deepening globalization (International Organization for Migration, 2017). The 53 countries of the WHO European Region are home to a population of nearly 920 million people, almost one-seventh of the world’s population. International migrants account for almost 10% (90.7 million) of the population living in the countries of the Region and 35% in the global migrant population (258 million) (World Health Organization, 2018). In the report of WHO from 2018, in the WHO European Region, almost 10% of the population of almost 920 million are international migrants, accounting for 35% of the global international migrant population. The scale of the phenomenon varies greatly. The percentage of international migrants, including refugees, in the Region’s member states ranged from more than 50% in Andorra and Monaco to less than 2% in Albania, Bosnia and Herzegovina, Poland and Romania (World Health Organization, 2018). It has changed dramatically, climate change and the increased number of conflicts mean increasing numbers of migrants. In 2020, the percentage of international migrants was 13.5% (100.8 million) and refugees and asylum seekers 5.0% (7.9 million) (World Health Organization, 2022).

Migrants vary in length of stay in a country, residence status, movement, and country of origin and reasons for migration (De Grande et al., 2014; International Organization for Migration, 2013; Straiton et al., 2014; Nielsen et al., 2012; Sanz et al., 2011; Dias et al., 2010; OECD/European Union, 2015). Consequently, migrant health is becoming a common topic of interest for all member states of the WHO European Region. WHO actions focus, among others, on promoting the health status of refugees and migrants. This includes promoting health, ensuring equal access to healthcare, evidence-informed practice, and information systems. Most attention regarding refugees and migrants is given to infectious diseases (World Health Organization, 2018).

As for immigrants with valid residence permits in Poland, 423,000 people held them in 2020 (Foreigners in Poland after, 2019). In this group, the majority were men, mainly immigrants from Ukraine and Belarus, Germany and Vietnam. People aged 20–39 who came to study at Polish universities predominated (Office for foreigners (UDSC)). The full-scale invasion of Ukraine in February 2022 resulted in mass migration of Ukrainians to Poland and other European countries. Data from June 2024 shows that the number of Ukrainian refugees residing in Poland is 957,504 (UNHCR).

With the influx of migrants, there are challenges for healthcare providers in providing healthcare, which include language and communication problems (De Vito et al., 2015), cultural, religious, worldview barriers (Chiu et al., 2003; Broom et al., 2019), lack of knowledge about healthcare eligibility among migrants and providers (International Organization for Migration, 2013; Simon et al., 2015), and administrative procedures in the country (Huddleston et al., 2015). Health professionals are often unsure whether migrants are eligible for health services (Burchill and Pevalin, 2012; Straßmayr et al., 2012). Studies show that immigrants are less likely to use health services and are more likely to receive lower quality healthcare than the general population (Alizadeh and Chavan, 2016; Schneider et al., 2002). A study of immigrant integration rates conducted in all EU and OECD countries found that differences between foreign-born and native-born individuals in their reported unmet medical needs were observed mainly in Central and Eastern European countries (e.g., Estonia and Poland), but also in countries that receive large numbers of refugees (e.g., Sweden) (OECD/European Union, 2015).

Problems in relations between medical personnel and immigrant patients are all the more serious the lower the level of cultural competence of medical personnel. This is because a high level of competence makes it possible to provide appropriate care to patients with different values, beliefs or behaviors. In addition to cultural competence, the attitude of staff toward patients plays an important role–in holistic medical care (Ferguson and Candib, 2002). Attitude is sometimes defined as the tendency to respond in a socially defined way. Attitudes have cognitive components (e.g., beliefs or knowledge), affective components (e.g., feelings or emotions), and behavioral components (e.g., a predisposition that may influence whether and how to act) (Marcinkowski and Reid, 2019). Studies confirm the presence of medical personnel’s biases against foreigners (Garcia et al., 2006; Van Ryn and Burke, 2000; Hudelson et al., 2010). Lack of knowledge and other cultural competencies, previous work experience, or stereotypes can adversely affect the relationship between healthcare workers and immigrants (Michaelsen et al., 2004; Hudelson et al., 2010; Hamilton and Essat, 2008; Suphanchaimat et al., 2015). According to study findings, a patient who perceives negative attitudes experiences low satisfaction with the services provided, exhibit passivity in the treatment process, or eventually limit the use of medical services in the host country (Scheppers et al., 2006), as well as achieve poor treatment outcomes or potentially face discriminatory or prejudicial treatment. The issue of cultural competence of healthcare providers is of big importance also in the context of aging societies, which also affects the characteristics of migrating populations. As it is found in the study by Makri and Giannouli (2022), culture has influence on differences regarding cognitive function, the emotional-social dimension, the perceptions and stereotypes that prevail, psychopathology-mental disorders, as well as at the level of suicides in the elderly. These findings indicate important aspect of health and social care on elderly coming from different cultures (Makri and Giannouli, 2022).

The Health of refugees from Ukraine in Poland 2022 survey showed that the most frequently mentioned obstacle to accessing healthcare was the information barrier (lack of information, language or cultural barrier). In general, the Polish healthcare system is required to ensure access to basic healthcare services for all, including migrants. Healthcare workers, often to a large extent, demonstrate professionalism and willingness to help, striving to provide appropriate care, regardless of the patient’s status. However, the availability and quality of services can be uneven. Migrants may encounter language, cultural and bureaucratic barriers that can make it difficult to use full healthcare. In addition, in some cases there may be difficulties in accessing appropriate services in emergencies or for those without health insurance (World Health Organization, 2023).

As refugees and migrants may face various challenges related to factors affecting their health, as well as healthcare difficulties–such as cultural barriers, financial, administrative, linguistic, cultural issues–health systems should develop long term policies and adjustments. Addressing the difficulties and cultural barriers, several Member States have introduced an intercultural mediator function to facilitate dialogue between patients and healthcare professionals (World Health Organization, 2018).

The issue of cultural competence of medical personnel has been discussed quite extensively in the literature. The research results in research tools for measuring this phenomenon. These include The Cultural Intelligence Scale (CQS), The Cross-Cultural Competence Inventory, The Healthcare Provider Cultural Competence Instrument (HPCCI), and the Cultural Competence Self Assessment Protocol for Healthcare Organizations and Systems (CCSAP) (Ang et al., 2007; Schwarz et al., 2015; Campinha-Bacote, 2003). Despite the potential impact of healthcare providers’ cultural attitudes toward immigrants, measurement tools in this area are lacking (Gozu et al., 2007). Therefore, the aim of the present study was to design and validate a questionnaire that allows for a relatively quick and easy assessment of the intensity and direction of health professionals’ attitudes toward immigrant patients. The main point is the possibility of carrying out correlational studies in which, thanks to a short scale, analyses can be made of the attitudes of healthcare workers toward immigrants with variables relating to the care provided, equal access to this care, satisfaction of immigrants with care, etc. This is important in monitoring the quality of healthcare and in planning changes in the professional education of healthcare workers.

## 2 Methods

### 2.1 Instrument development

The Attitudes of Health Professionals Towards Immigrants (AHPI) questionnaire was constructed to measure the intensity and direction of health professionals’ attitudes toward immigrant patients. Work on the questionnaire began with the preparation of a list of statements with which to characterize the attitudes of health professionals toward immigrant patients. For this purpose, reference was made to the knowledge and experience of healthcare professionals, psychology, sociology and academics experts in culturally sensitive medical care issues, as well as an analysis of the literature on the subject. Research on healthcare workers’ attitudes toward migrants (Garcia et al., 2006; Van Ryn and Burke, 2000; Hudelson et al., 2010; Gozu et al., 2007; Dias et al., 2012; Pitkänen and Kouki, 2002) and conceptual reports on attitudes and behavior (Allport, 1954; Kraus, 1995a; Kraus, 1995b; Kim and Hunter, 1993a; Kim and Hunter, 1993b; Brown and Lopez, 2001), were reviewed. The list included statements related to three components of attitude: emotional, cognitive and behavioral components. Sixty-seven statements were extracted, with 17 of them rejected already in the initial selection, mainly for linguistic reasons (they could lead to misunderstandings). Content relevance was assessed in the second stage. In the first stage, 10 knowledgeable judges (5 academics and 5 practitioners: 3 doctors and 2 nurses) were invited to evaluate the test items on a three-point scale: 1 – the item is essential to the test, 2 – the item is useful but not essential, 3 – the item should not be within the test. The averages for each statement were then calculated. After analysis, statements that were considered essential to the scale by less than half of the judges were rejected. These statements received an average of less than 1.5 (Hornowska, 2005). After all 31 items were rejected. The remaining 19 items were subjected to further evaluation by competent judges (content validation is described in the results). The final version of the AHPI consists of 7 statements relating to 3 attitude components to which the respondent must respond. Assessment of the extent to which the respondent agrees with a given statement is made on a 5-point Likert-type scale, according to the accepted scoring: 1- strongly disagree, 2 – disagree; 3 – hard to say; 4 – agree; 5 – strongly agree. The total score of the scale is the sum of the individual items. The maximum score on the scale is 35 points, while the minimum score is 5 points. The higher the score, the more positive the attitude toward immigrant patients.

### 2.2 Study design and participants

This study was based on a cross-sectional survey conducted between October 2019 and September 2020. The study sample consisted of healthcare workers employed in hospitals in central and eastern Poland. Hospital units were randomly drawn (units with I and II reference levels). The study was open to those who had professional contact with immigrants (inclusion criterion). By contact with immigrants, it was meant the performance of professional activities with immigrants in the workplace. Finally, psychometric properties and descriptive statistics were developed based on the results obtained in a group of 347 participants (246 women, 101 men) aged 25–58 years. The mean age of the participants was 38.03 (SD = 8.84). The participants in the study were those working in the healthcare system: midwives [n = 120; 34.6%], nurses [n = 120; 34.6%], doctors [n = 107; 30.8%] (Table 1).

**TABLE 1:** Characteristics of the study group.

Variable		[N = 347]	
		n	%
Sex	Female	179	51.6
	Male	165	47.6
	Other	3	0.9
Education	Vocational	10	2.9
	Secondary	126	36.3
	Higher	211	60.8
Place of residence	Rural	106	30.5
	Urban	241	69.5
Marital status	Married	295	85.0
	Single	34	9.8
	Widowed/widowered	7	2.0
	Divorcee/divorcee	11	3.2
Relationship to faith	Believer	272	78.4
	Non-practicing believer	61	17.6
	Non-believer	14	4.0

### 2.3 Data collection

Prior to distributing the survey, the purpose of the research was explained and participants were informed that involvement was voluntary and anonymous. Surveys were completed anonymously via pencil and paper. Upon completion, participants sealed their responses in an envelope and returned it to a trained research assistant. In addition, healthcare workers can eave their responses in a box placed in the secretary’s office. Participants were made aware they could withdraw from the study at any time without penalty. The research assistants then delivered the sealed envelopes to the lead researcher for data analysis. No pressure was applied on respondents; they were left free to ask questions and to obtain explanations.

At the beginning of the questionnaire, a specific statement was reported specifying that filling in the questionnaire was understood as consent to take part in the study. During the data collection a researcher was available if any doubts or questions emerged. Data were stored in a secure, encrypted database available only to the research team. No personal data were collected. The data presented in this study is available on request from the corresponding author.

### 2.4 Ethical issues

All procedures were approved by the Ethical Committee of the Institute of Psychology, The John Paul II Catholic University of Lublin prior to data collection. Steps were taken throughout the process to maintain participant confidentiality and adhere to ethical guidelines.

### 2.5 Statistical analysis

Content relevance was first evaluated on a database of data from 10 competent judges. The content relevance index (CVI) and Kappa coefficient were used, which provide a better understanding of content relevance because they eliminate any random agreement (Straßmayr et al., 2012; Kim and Hunter, 1993a). For the relevance scale, a four-point Likert scale was used, and responses include: 1 = not relevant, 2 = somewhat relevant, 3 = somewhat relevant, and 4 = very relevant. CVI was calculated using the Item-CVI (I-CVI) index. I-CVI is the percentage of content experts who gave an item a significance rating of 3 or 4 (agreed items/number of experts).

Next, descriptive statistics and correlations between scale items were analyzed to assess the psychometric properties of the questionnaire items. Exploratory factor analysis made it possible to check whether there were grounds for extracting latent structures on the basis of observed correlations between observable variables. Bartlett’s sphericity test of the correlation matrix, the Kaiser Meyer-Olkin statistic, and the percentage of variance of the indicators forming the scale - reproduced by the first principal component - were used to assess the validity of extracting hidden factors. The factor structure was verified using confirmatory factor analysis with maximum likelihood estimation. In the analysis of the fit of the models tested, the following measures of fit were taken into account: RMSEA, PCLOSE, CFI, AGFI, GFI. The reliability of the scales (internal consistency and absolute stability) was also analyzed.

Criterion validity was assessed by comparing the score of the AHPI with the score Illegal Immigrant Scale (IIS). The scale includes 20 statements to which participants responded with how strongly they agreed with each one (from agree strongly to disagree strongly). The original scale uses the “illegal” label, so we changed the statements to “immigrants” so that the scale did not differentially match or mismatch across our label conditions. Statements ranged from social (“Immigrants should not be discriminated against”) to political (“Immigrants who give birth to children in Poland should be made citizens”) Scores were computed by averaging the responses after reverse-coding the relevant item (Ommundsen et al., 2014).

Analyses were performed using IBM SPSS 25 and IBM SPSS Statistic Amos. (IBM Corp., 2012; Armonk, NY, USA).

## 3 Results

### 3.1 Content validity

The I-CVI values for 7 items were above 0.8. The Kappa coefficient value of 0.72 indicates satisfactory consistency.

### 3.2 Construct validity

**FIGURE 1:**
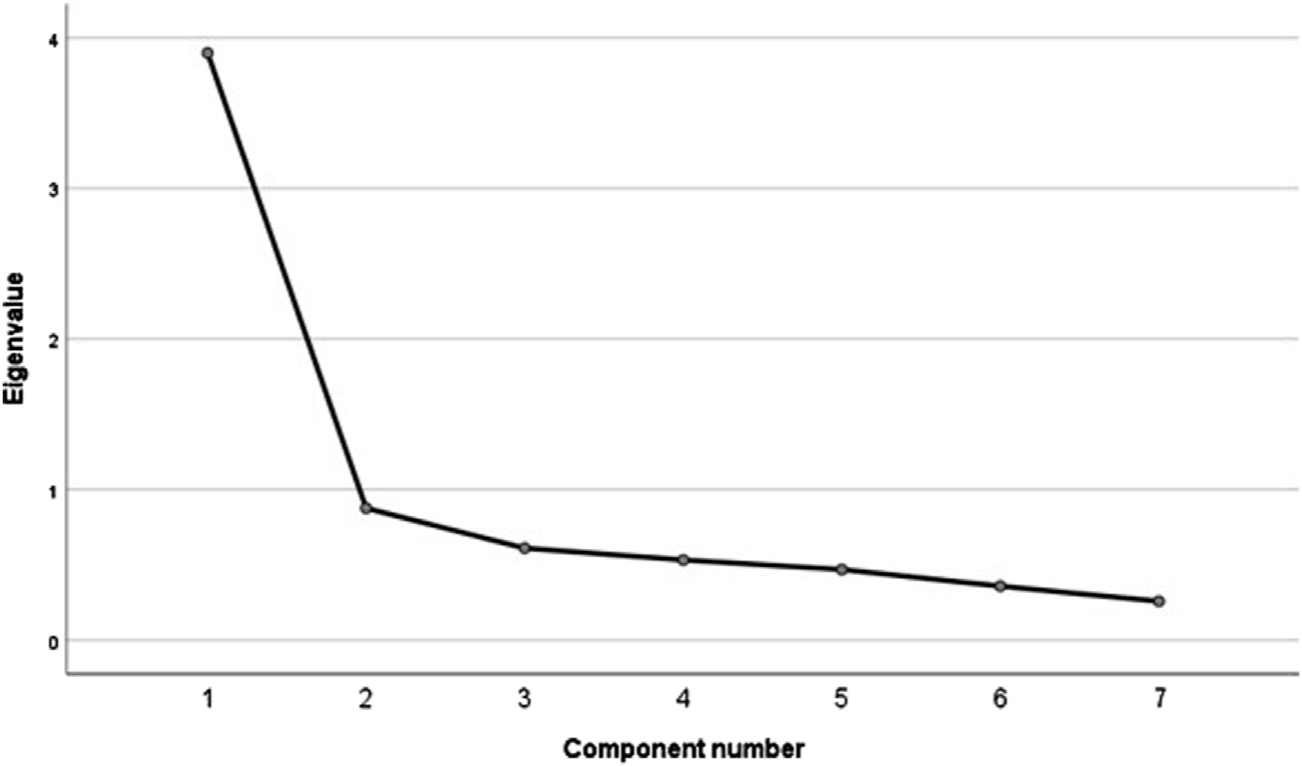
Screen plot with the exploratory factor analysis (EFA).

Factor relevance was assessed using two methods: exploratory factor analysis (EFA) and confirmatory factor analysis (CFA). EFA was conducted using the principal components method. Oblimin rotation was used with a degree of diagonal delta = 0 (non-orthogonality of factors was assumed). The validity of the choice of factor analysis model was formally confirmed using the Kaiser-Meyer-Olkin (KMO) index (0.87) and Bartlett’s sphericity test (χ2 = 1,042.53; p< 0.001). The values of the Bartlett test statistic allow us to conclude that the correlation matrices are not unitary matrices for all analyzed scales. In turn, the high values of the Kaiser-Meyer-Olkin statistic indicate that the observed correlation matrices are the product of the interaction of common factors. EFA allows us to assume a univariate solution, which explains 55.70% of the variance. The values of the factor loadings ranged from 0.52 to 0.84 (Table 2). The one-factor solution is confirmed by the analysis of the scree plot (Figure 1).

**TABLE 2:** Factor loadings.

Items	Factor loading
I feel positive emotions about providing healthcare to immigrant patients	0.829
I have a positive attitude toward the use of medical care in my country by immigrant patients	0.807
I have a belief that the healthcare provided to immigrant patients brings me many benefits	0.842
I encounter positive emotions toward immigrant patients in my workplace	0.675
I try to take into account the preferences of immigrant patients in the healthcare they receive	0.738
I support facilitating access to medical care in my country for immigrant patients	0.761
I try to create a friendly atmosphere in my relationship with immigrant patients	0.521

**FIGURE 2:**
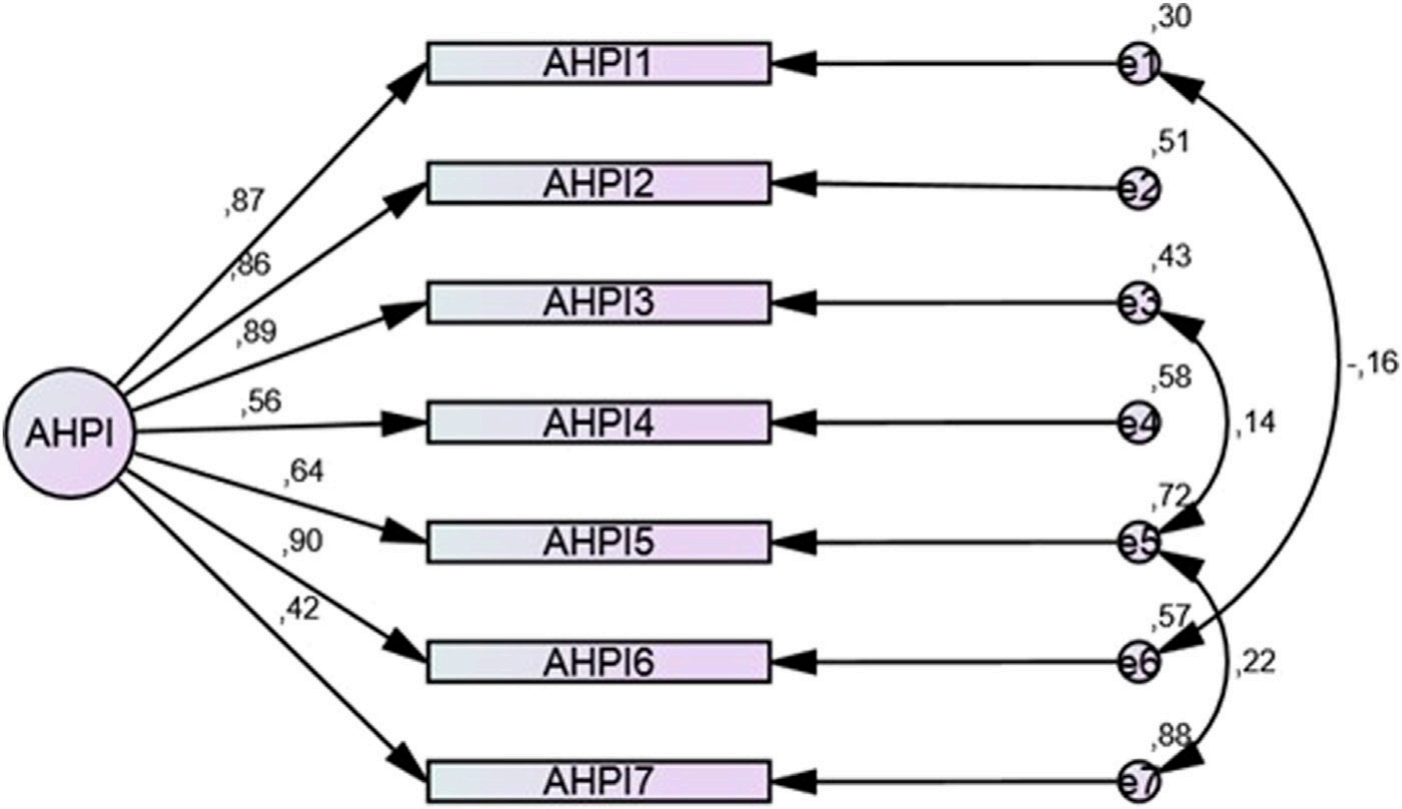
Path diagram with summary of the confirmatory factor analysis (CFA).

A CFA was conducted to verify the proposed model. It was assumed that measurement errors for individual test items could be correlated with each other. The univariate model without correlation of measurement errors was a poor fit, with a high chi-square statistic value and RMSEA (root mean square error of approximation) exceeding 0.1. The analysis allowing for correlation shows that the univariate model is a good fit to the data (Figure 2). The chi-square test statistic was found to be statistically insignificant (χ2 = 12.085; *p* = 0.147). Other fit measures were also referred to, with acceptable values indicating a good model fit: RMSEA = 0.038; PCLOSE = 0.624; AGFI = 0.966; GFI = 0.990; CFI = 0.996. A CFA was also conducted by gender to assess how this variable may affect the interpretation of the items differently. The results for men were satisfactory: χ2 = 6.335; *p* = 0.850; RMSEA = 0.001; PCLOSE = 0.959; AGFI = 0.972; GFI = 0.989; CFI = 1.000. In the women’s group the results were also satisfactory: χ2 = 9.617; *p* = 0.565; RMSEA = 0.001; PCLOSE = 0.840; AGFI = 0.961; GFI = 0.985; CFI = 1.000.

### 3.3 Criterion validity

The overall score of the AHPI questionnaire correlated moderately and statistically significantly with the score of the Illegal Immigrant Scale (IIS) [r – 0.472]. The more positive the attitude towards immigration, the more positive the attitude towards immigrant patients.

### 3.4 Internal consistency. Test–retest reliability

The reliability of the scale was 0.86 [95% CI = 0.84–0.88]. It is assumed that for the scale to be considered reliable, Cronbach’s α coefficient should be greater than 0.6. The result obtained indicates satisfactory consistency of the method and allows it to be used in research. Cronbach’s α coefficient only increases slightly after removing statement 7. The discriminatory power of the items is satisfactory. Correlations of individual statements with the total AHPI scale score ranged from 0.41 (statement 7) to 0.75 (statement 3) (Table 3). Reliability testing based on the test-retest method (after 3 weeks) showed high test-retest reliability (0.75).

**TABLE 3:** Cronbach’s alpha reliability coefficients.

Items	Item correlation Total	Cronbach’s alpha after removing items
I feel positive emotions about providing healthcare to immigrant patients	0.737	0.830
I have a positive attitude toward the use of medical care in my country by immigrant patients	0.706	0.834
I have a belief that the healthcare provided to immigrant patients brings me many benefits	0.748	0.828
I encounter positive emotions toward immigrant patients in my workplace	0.557	0.854
I try to take into account the preferences of immigrant patients in the healthcare they receive	0.636	0.844
I support facilitating access to medical care in my country for immigrant patients	0.650	0.843
I try to create a friendly atmosphere in my relationship with immigrant patients	0.412	0.873

### 3.5 Descriptive statistics

Descriptive statistics for each item are shown in Table 4. Respondents scored highest on item 7: “I try to create a friendly atmosphere in relations with immigrant patients”, and lowest on item 6: “I support facilitating access to medical care in my country for immigrant patients”. The overall score of the scale is close to the median value of 17.5 (M = 18.56), suggesting no dominance of negative or positive attitudes. The distributions of the individual items and the overall score were not close to a normal distribution.

**TABLE 4:** Descriptive statistics.

Item number	M	Me	SD	Skewness	Kurtosis	Min	Max
Item 1	2.63	3.00	1.03	−0.09	−0.35	1.00	5.00
Item 2	2.36	2.00	1.12	0.38	−0.69	1.00	5.00
Item 3	2.52	3.00	1.11	0.13	−0.77	1.00	5.00
Item 4	2.41	3.00	0.94	0.07	−0.36	1.00	5.00
Item 5	3.02	3.00	1.06	−0.37	−0.44	1.00	5.00
Item 6	2.21	2.00	1.17	0.53	−0.76	1.00	5.00
Item 7	3.43	4.00	1.03	−0.64	0.32	1.00	5.00
Global score	18.56	19.00	5.54	−0.10	−0.33	7.00	33.00

## 4 Discussion

The AHPI questionnaire can be a useful tool for assessing the attitudes of healthcare workers toward immigrant patients. Exploratory factor analysis made it possible to isolate one factor. The results of confirmatory factor analysis confirmed the accuracy of the adopted model. The results of the reliability analysis of the tool were satisfactory. The tool can be used to assess staff attitudes toward immigrant patients. The items in the questionnaire touched on three components of attitude: emotional, cognitive and behavioral. With such a diagnosis, it is possible to take effective measures in the area of cultural competence, for example, with regard to sensitivity to cultural difference. The content validity of the AHPI was established through expert review by 10 judges. The Item-level Content Validity Index (I-CVI) values for all 7 items exceeded the recommended threshold of 0.78 for relevance with 10 experts (Lynn, 1986). This suggests the 7 items adequately represent the domain of healthcare provider attitudes toward immigrant patients. Additionally, the scale-level Content Validity Index (S-CVI) was 0.92, well above the recommended 0.80 threshold, further supporting the overall content validity of the AHPI (Polit et al., 2007). The construct validity was examined using both exploratory factor analysis (EFA) and confirmatory factor analysis (CFA). Bartlett’s test of sphericity was significant and the KMO value was 0.87, indicating the data was suitable for EFA. The EFA revealed a unidimensional factor structure explaining 55.7% of the variance. Factor loadings ranged from 0.52 to 0.84, all above the minimum threshold of 0.40 for a sample size of 300 (Hair et al., 2010). CFA was conducted to verify the one-factor structure obtained in the EFA. Allowing for correlated measurement errors between items significantly improved model fit. The fit indices (CFI = 0.996, RMSEA = 0.038, SRMR = 0.022) met recommended cut-offs, demonstrating good fit (Hu and Bentler, 1999). This confirms the hypothesized unidimensional structure of the AHPI measuring healthcare provider attitudes. Overall, the EFA and CFA provide initial evidence for the construct validity of the AHPI based on its internal factor structure. Further validation in more diverse healthcare provider samples is needed to establish generalizability. Internal consistency was estimated using Cronbach’s alpha, which was 0.86, surpassing the 0.70 threshold for adequate reliability in early scale development (Nunnally and Bernstein, 1994). Inter-item correlations ranged from 0.25 to 0.60, within the optimal range of 0.20–0.60 for a unidimensional scale (Clark and Watson, 1995). Test-retest reliability over a 3-week interval was 0.75, which can be considered adequate given the potential for attitudes to change over time (Wang et al., 2017). A test-retest value between 0.70–0.80 is considered acceptable (Terwee et al., 2007). The questionnaire has a simple design, and the questions are at a high level of generality. The authors assumed that with this approach it would be possible to apply the tool in different socio-cultural or religious settings. The valid tool that can be used to assess and monitor the attitudes of healthcare providers towards immigrants, offering potential insights for improving care and reducing disparities in healthcare access and quality for immigrant populations. The use of the AHPI in practice has potential benefits for healthcare professionals. Understanding attitudes towards immigrant patients allows for the introduction of appropriate interventions that can improve relationships between healthcare professionals and patients and improve the overall quality of services provided. The results obtained from this tool can be used to design training programs that aim to increase awareness and knowledge about caring for patients from different cultural backgrounds. Regular use of this tool allows for monitoring changes in healthcare professionals’ attitudes over time. This can be particularly useful in the context of assessing the effectiveness of implemented training programs. Understanding how healthcare professionals’ attitudes affect the accessibility and quality of services for immigrants can lead to better adaptation of policies to the needs of this group.

In general, building tools to study attitudes involves certain limitations. The vague semantic field of the concept of attitude poses enormous problems for the researcher in constructing accurate research tools. Despite the widespread adoption of structural (cognitive, emotional and behavioral components) definitions of attitude, researchers are usually limited to measuring one of its components and, on the basis of such measurement, formulate conclusions about the “whole” attitude. In addition, attitude as a mental phenomenon is not subject to direct observation, so its study may be based only on the measurement of what we consider to be the manifestation of a particular attitude (e.g., the lack of a relationship between the attitude studied and the reactions recorded) (Schahbasi et al., 2021). It is also worth remembering that self-reporting methods are not free from the influence of social approval, the desire to present oneself in a more favorable light.

In conclusion, this initial validation study provides support for the reliability and validity of the 7-item AHPI questionnaire to measure healthcare provider attitudes toward immigrant patients. The AHPI has potential as a research tool, however further research in larger and more diverse healthcare provider populations is required to confirm the psychometric properties of the AHPI. Ongoing research should examine the factor structure, assess the risk of social desirability bias, evaluate responsiveness and sensitivity to change, and establish scores that can differentiate between positive and negative attitudes. Although the tool was tested for gender, other relevant sociodemographic variables such as age, work experience, level of education or medical specialization may also influence attitudes and were not sufficiently taken into account. It is also worth considering the specifics of the different work environments that healthcare workers may operate in. Attitudes may be influenced by factors such as workload or institutional support.

## Data Availability

Publicly available datasets were analyzed in this study. This data can be found here: 10.6084/m9.figshare.24047130.v1.
