# Supplementary material for: Design and validation of a questionnaire to measure the attitudes of health professionals towards immigrants (AHPI)
Source: Front Pharmacol. 2024 Oct 10;15:1287536. doi: 10.3389/fphar.2024.1287536 (PMC11499901; doi:10.3389/fphar.2024.1287536)
Supplement: Supplementary file 1 [file DataSheet2.PDF]

Kwestionariusz postawy pracowników służby zdrowia wobec imigrantów

Poniżej znajduje się siedem stwierdzeń dotyczących różnych aspektów opieki zdrowotnej sprawowanej nad imigrantami, z którymi możesz się zgodzić lub nie. Używając poniższej skali od 1 do 5 wskaż, w jakim stopniu zgadzasz się z każdym stwierdzeniem, wstawiają co odpowiednią cyfrę w polu za danym stwierdzeniem.

1 – Zdecydowanie nie zgadzam się

2 – Nie zgadzam się

3 – Ani zgadzam się, ani nie zgadzam się

4 – Zgadzam się

5 – Zdecydowanie zgadzam się

Odczuwam pozytywne emocje w związku ze sprawowaniem opieki zdrowotnej nad pacjentami imigrantami	______
Mam pozytywny stosunek do korzystania z opieki medycznej w moim kraju przez pacjentów imigrantów	______
Mam przekonanie, że opieka zdrowotna sprawowana nad pacjentami imigrantami przynosi mi wiele korzyści	______
W moim miejscu pracy spotykam się z pozytywnymi emocjami wobec pacjentów imigrantów	______
Staram się uwzględniać w sprawowanej opiece zdrowotnej nad pacjentami imigrantami ich preferencje	______
Popieram ułatwienia w dostępie do opieki medycznej w moim kraju dla pacjentów imigrantów	______
Staram się tworzyć przyjazną atmosferę w relacjach z pacjentami imigrantami	______
