# Supplementary material for: Design and validation of a questionnaire to measure the attitudes of health professionals towards immigrants (AHPI)
Source: Front Pharmacol. 2024 Oct 10;15:1287536. doi: 10.3389/fphar.2024.1287536 (PMC11499901; doi:10.3389/fphar.2024.1287536)
Supplement: Supplementary file 2 [file DataSheet1.PDF]

**The Attitudes of Health Professionals Towards Immigrants (AHPI)**

Below are seven statements about various aspects of health care provided to immigrants that you may agree or disagree with. Using the scale from 1 to 5 below, indicate to what extent you agree with each statement by putting the appropriate number in the box behind the statement.

1- strongly disagree,
2- disagree;
3- hard to say;
4- agree;
5- strongly agree

I feel positive emotions about providing healthcare to immigrant patients ____________

I have a positive attitude toward the use of medical care in my country by immigrant patients ____________

I have a belief that the healthcare provided to immigrant patients brings me many benefits ____________

I encounter positive emotions toward immigrant patients in my workplace ____________

I try to take into account the preferences of immigrant patients in the healthcare they receive ____________

I support facilitating access to medical care in my country for immigrant patients ____________

I try to create a friendly atmosphere in my relationship with immigrant patients ____________
